# MicroRNA-17-5p induces drug resistance and invasion of ovarian carcinoma cells by targeting PTEN signaling

**DOI:** 10.1186/s40709-015-0035-2

**Published:** 2015-10-22

**Authors:** Ying Fang, Changyan Xu, Yan Fu

**Affiliations:** Department of Gynecology, The First Hospital of Jilin University, Changchun, Jilin People’s Republic of China; Department of Gynecology, No. 208 Hospital of Chinese People’s Liberation Army, Changchun, Jilin People’s Republic of China; Department of Medical Administration, The First Hospital of Jilin University, Changchun, Jilin People’s Republic of China

**Keywords:** AKT, Apoptosis, PTEN, Metastasis, miRNAs, miRNA-17-5p, EMT

## Abstract

**Background:**

The miR-17-5p was overexpressed in ovarian cancer cells, and those cells were treated with paclitaxel. The proliferation of ovarian cancer cells was assessed by MTT assay. The Caspase-Glo3/7 and TUNEL assay were used to examine the effect of miR-17-5p on paclitaxel-induced apoptosis in ovarian cancer cells. The migration and invasion of ovarian cancer cells were analyzed by BD matrigel assays. Western blot was performed to evaluate the expression of apoptotic proteins and epithelial-mesenchymal transition markers in ovarian cancer cells.

**Results:**

The survival rate of ovarian cancer cells was increased after overexpression of miR-17-5p. The apoptosis decreased in miR-17-5p overexpressed ovarian cancer cells. Altered miR-17-5p expression affected migration and invasion of ovarian cancer cells. The overexpression of miR-17-5p altered the expression of EMT markers. miR-17-5p activates AKT by downregulation of PTEN in ovarian cancer cells.

**Conclusion:**

Our results indicate that miR-17-5p might serve as potential molecular therapeutic target.

## Background

Ovarian cancer accounts for about 3 % of cancers among women in USA. It is estimated that about 21,290 women will receive a new diagnosis of ovarian cancer and about 14,180 women will die from ovarian cancer in 2015, even though the rate at which women are diagnosed with ovarian cancer has been slowly falling over the past 20 years. Only 20 % of ovarian cancers are found at an early stage and more than 90 % of patients live longer than 5 years if ovarian cancer can be found at early stage [1]. The most often used tests to screen for ovarian cancer are transvaginal ultrasound and the CA-125 blood test. However, the sensitivity and specificity of transvaginal ultrasound and the CA-125 blood test are poor [2, 3]. Chemotherapy is the primary mode of treatment for patients with ovarian cancer. However, the treatment failure is high due to resistance [4]. Therefore, it is important to investigate the molecular mechanisms and identify valuable predictive markers in ovarian cancer.

MicroRNAs (miRNAs) are a family of small non-coding RNAs that are 20–22 nucleotides in length. Studies have demonstrated that miRNAs regulate the expression of target genes at the post-transcriptional level and play important roles in the tissue-specific protein expression. An increasing number of studies have reported that miRNAs play important roles in tumorigenesis, progression, diagnosis and prognosis of ovarian cancer [5]. The expression level of miRNAs is different in ovarian cancer as demonstrated by miRNA expression profiling studies [6, 7]. For example, miR-200c and miR-31 play important roles in ovarian cancer metastasis [8]. Recent studies have reported that miRNAs can be used as prognostic biomarkers in ovarian cancer [9, 10]. Also, it has been reported that some serum miRNAs could serve as biomarkers in ovarian cancer [11].

In the present study, we aimed to examine the role of miR-17-5p in ovarian cancer. We found that overexpression of miR-17-5p induces drug resistance, migration and invasion of ovarian cancer cells. Importantly, miR-17-5p enhanced Epithelial-Mesenchymal Transition (EMT) of ovarian cancer cells by targeting PTEN signaling. Our findings indicate that miR-17-5p might serve as a potential biomarker to predict the treatment and be targeted for novel therapeutic strategies.

## Results

### Overexpression of miR-17-5p induces drug resistance of ovarian cancer cells

**Fig. 1:**
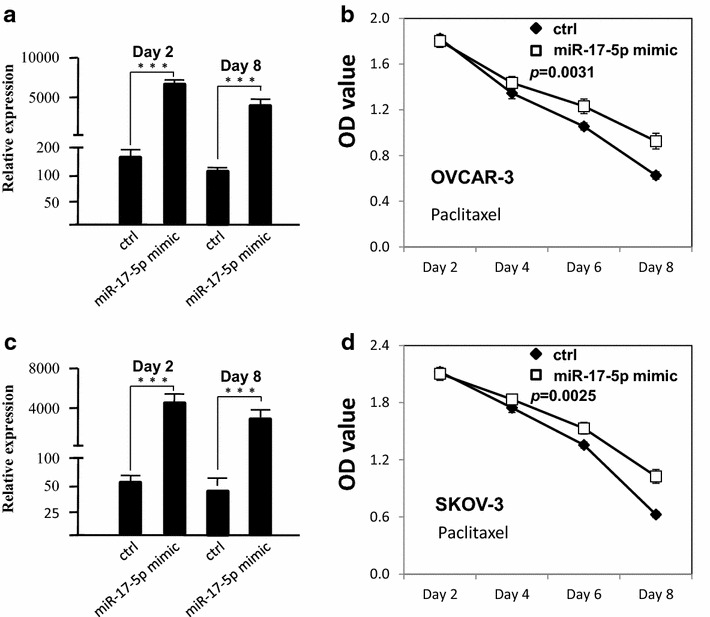
miR-17-5P decreased the chemo-sensitivity of ovarian cancer cells. **a** The miR-17-5p expression level was examined by qRT-PCR after transfection in OVCAR-3 cells. **b** The proliferation of OVCAR-3 cells after paclitaxel treatment (6 µmol L^−1^) for 8 days. **c** The miR-17-5p expression level was examined by qRT-PCR after transfection in SKOV-3 cells. **d** The proliferation of SKOV-3 cells after paclitaxel treatment (6 µmol L^−1^) for 8 days. All experiments were performed three times in triplicate

To examine the function of miR-17-5p on proliferation of ovarian cancer cells after paclitaxel treatment, miR-17-5p was overexpressed in ovarian cancer cells with Lipofectamine 2000 and the cell survival rate was measured by MTT assay. As shown in Fig. 1a, d, the miR-17-5p expression level was significantly increased in both OVCAR-3 and SKOV-3 cells after transfection. The survival rate of OVCAR-3 and SKOV-3 cells was increased after overexpression of miR-17-5p when ovarian cancer cells were treated with paclitaxel, compared to the negative control group (Fig. 1b, d, *p* = 0.0025). The IC_50_ was 6.1 ± 1.1 µmol L^−1^ in control group, and the IC_50_ was 8.2 ± 0.8 µmol L^−1^ in miR-17-5p mimic group.

### miR-17-5p decreases apoptosis of ovarian cancer cells

**Fig. 2:**
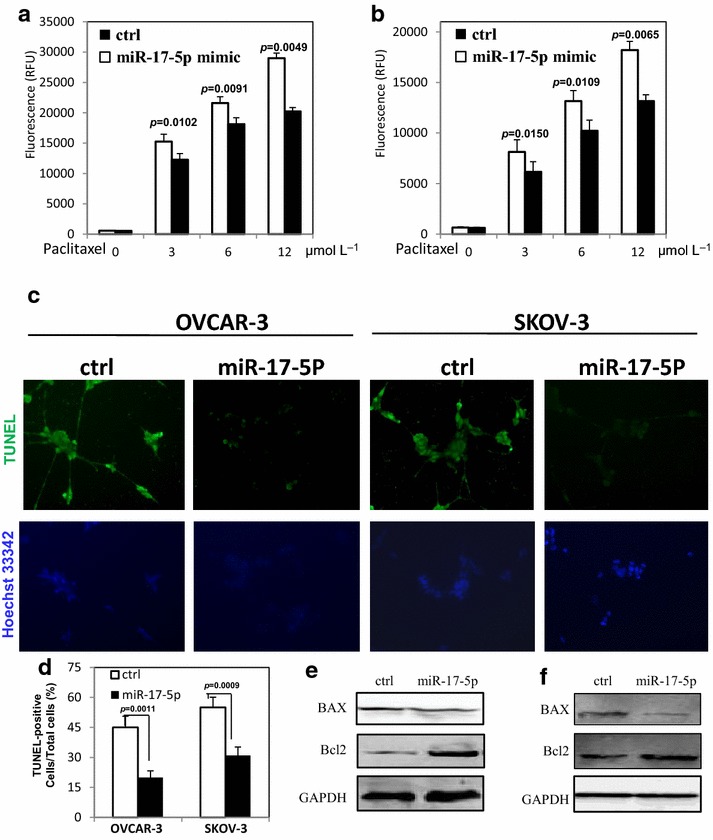
miR-17-5p inhibits apoptosis in ovarian cancer cells after paclitaxel treatment. **a**, **b** The caspase 3/7 activity was decreased in OVCAR-3 and SKOV-3 cells transfected with miR-17-5p after paclitaxel treatment. **c** The TUNEL positive cells after OVCAR-3 and SKOV-3 cells were treated with paclitaxel. **d** The quantification of the percentage of TUNEL-positive cells. **e** The apoptotic proteins level in OVCAR-3 cells transfected with miR-17-5p mimic after paclitaxel treatment. **f** The apoptotic proteins level in SKOV-3 cells transfected with miR-17-5p mimic after paclitaxel treatment. All experiments were performed three times in triplicate

OVCAR-3 and SKOV-3 cells were used to examine the effect of miR-17-5p on chemotherapy-induced apoptosis by treating with different doses of paclitaxel after overexpression of miR-17-5p. We found that the miR-17-5p reduced the sensitivity of ovarian cancer cells to the effects of paclitaxel by inhibiting the caspase 3/7 activities (Fig. 2a, b). The TUNEL assay also showed that the number of apoptotic cells in miR-17-5p overexpressed ovarian cancer cells was less than that in control group (Fig. 2c, d). We further analyzed the expression of apoptotic related proteins by western blot after paclitaxel treatment. As shown in Fig. 2e, f, the overexpression of miR-17-5p inhibited the ability of chemotherapy to increase BAX expression, while the Bcl-2 expression was increased in miR-17-5p overexpressed ovarian cancer cells.

### miR-17-5p promotes migration and invasion in ovarian cancer cells

**Fig. 3:**
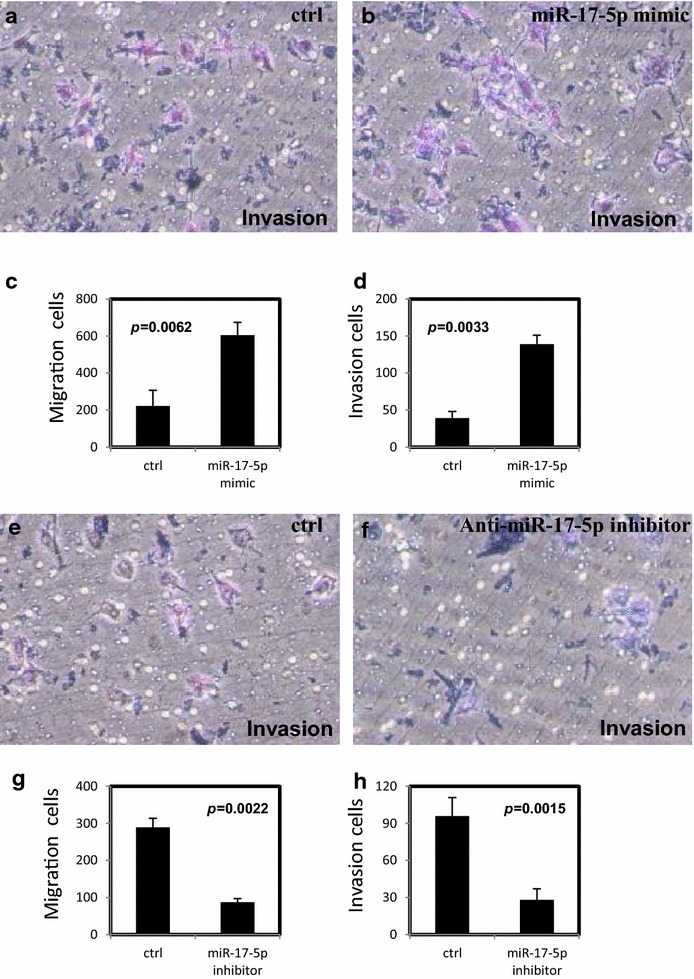
miR-17-5P increases migration and invasion of ovarian cancer cells. **a**–**d** miR-17-5p increased migration and invasion in OVCAR-3 cells after transfection with miR-17-5p mimic. **e**–**h** The migration and invasion were decreased in OVCAR-3 cells after transfection with anti-miR-17-5p inhibitor

**Fig. 4:**
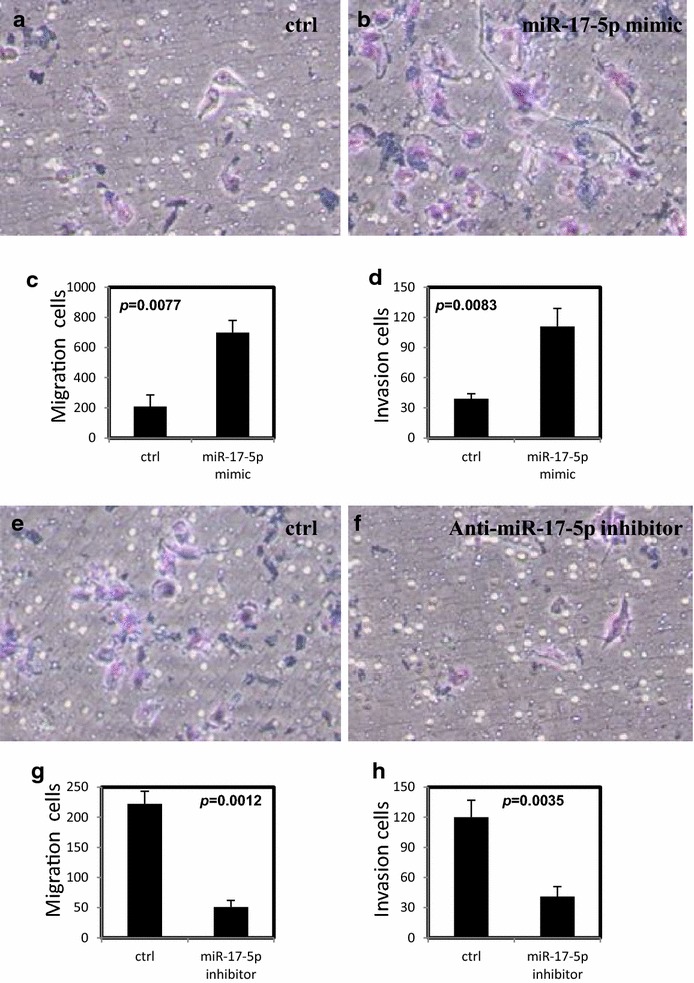
miR-17-5P increases migration and invasion of ovarian cancer cells. **a**–**d** miR-17-5p increased migration and invasion in OVCAR-3 cells after transfection with miR-17-5p mimic. **e**–**h** The migration and invasion were decreased in SKOV-3 cells after transfection with anti-miR-17-5p inhibitor. All experiments were performed three times in triplicate

We further assessed the effect of miR-17-5p on migration and invasion of ovarian cancer cells with BD transwell migration and matrigel invasion assays. We found that migration and invasion of OVCAR-3 cells (Fig. 3a–d) and SKOV-3 cells (Fig. 4a–d) were enhanced after transfection with miR-17-5p mimic. In contrast, the migration and invasion of OVCAR-3 cells (Fig. 3e–h) and SKOV-3 cells (Fig. 4e–h) were decreased with anti-miR-17-5p inhibitor treatment.

### miR-17-5p affects the expression of EMT markers and activates AKT in ovarian cancer cells

**Fig. 5:**
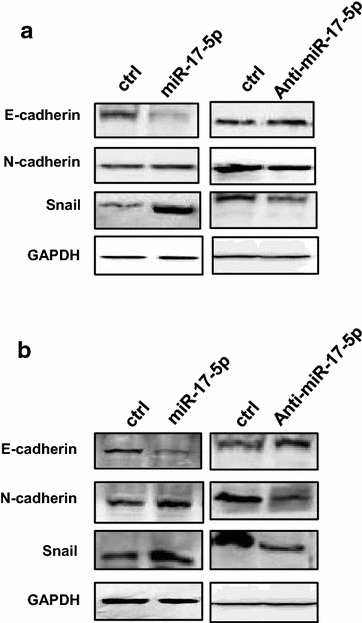
miR-17-5p regulates EMT markers expression in ovarian cancer cells. **a** The EMT markers level in OVCAR-3 cell after transfection with miR-17-5p mimic or anti-miR-17-5p inhibitor. **b** The EMT markers level in SKOV-3 cells after transfection with miR-17-5p mimic or anti-miR-17-5p inhibitor

**Fig. 6:**
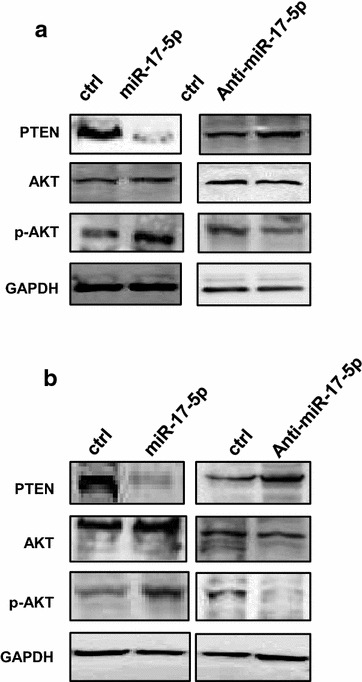
miR-17-5p activates AKT expression by regulating PTEN in ovarian cancer cells. **a** The expression of AKT and PTEN in OVCAR-3 cells after transfection with miR-17-5p mimic or anti-miR-17-5p inhibitor. **b** The expression of PTEN and AKT in SKOV-3 cells after transfection with miR-17-5p mimic or anti-miR-17-5p inhibitor

The expression of EMT markers was further examined by western blot after ovarian cancer cells were transfected with either miR-17-5p mimic or anti-miR-17-5p inhibitor. We found that the level of E-cadherin expression was significantly decreased, and the expression of N-cadherin, Snail and Vimentin were increased in both OVCAR-3 and SKOV-3 cells after overexpression of miR-17-5p (Fig. 5a). In contrast, increased E-cadherin expression and decreased N-cadherin, Snail and Vimentin were observed in OVCAR-3 and SKOV-3 cells transfected with anti-miR-17-5p inhibitor (Fig. 5a). Interestingly, we further found that the expression of p-AKT was increased, and the PTEN expression level was decreased in OVCAR-3 (Fig. 6a) and SKOV-3 cells (Fig. 6b) after transfection with miR-17-5p mimc. The transfection with anti-miR-17-5p inhibitor decreased p-AKT protein level and increased PTEN expression in OVCAR-3 (Fig. 6a) and SKOV-3 cells (Fig. 6b).

## Discussion

Growing evidence has demonstrated that miRNA expression correlates with tissue type, differentiation, aggression, response to therapy and prognosis [12, 13]. Studies have shown that miRNA act as oncogenes or tumor suppressors in variety types of tumors [14, 15]. Li et al. has reported that miR-17-5p regulates cell cycle and apoptosis in ovarian cancer tissues and serum of ovarian cancer patients [16]. miR-17-5p has been found to be expressed differentially in the serum of cancer tissue compared with that of non-cancerous tissues [17]. Studies have found that miR-17-5p is a key regulator of the G1/S phase cell cycle transition [18]. In our study, we demonstrated that the overexpression of miR-17-5p promoted the proliferation of ovarian cancer cells treated with paclitaxel. These results indicate that miR-17-5p induces drug resistance of ovarian cancer cells. By analysis of caspase 3/7 activity and TUNEL assay, we found that the overexpression of miR-17-5p decreases the paclitaxel-induced apoptosis of ovarian cancer cells. Meanwhile, the increased Bcl-2 and decreased BAX expression levels in miR-17-5p overexpressed ovarian cancer cells confirm that miR-17-5p decreases the paclitaxel-induced apoptosis of ovarian cancer cells. These results indicate that miR-17-5p might play an important role in conferring chemosensitivity to ovarian cancer cells.

Chemotherapy drugs are most effective when given in combination. Paclitaxel is often combined with other chemotherapy drugs, such as cisplatin. Therefore, further study is required to examine the function of miR-17-5p in combination of chemotherapy treatment. Ovarian cancer has a higher incidence of distant metastasis [19]. Once metastasis occurs, it becomes an incurable disease with limited survival time [20]. Metastasis is a complex, multistep process by which tumor cells disseminate from their primary site and form secondary tumors at a distant site [21]. Epithelial–mesenchymal transition (EMT) has been shown to play a critical role in promoting metastasis. EMT is a biological process that allows epithelial cells to lose their epithelial characteristics and acquire a mesenchymal phenotype [22]. EMT plays a critical role in ovarian cancer metastasis. Many miRNAs, such as miR-7 [23], miRNA-150 [24], miR-200c [25], modulate the EMT and metastasis of ovarian cancer cells. We showed that miR-17-5p increased migration and invasion in both OVCAR-3 and SKOV-3 cells after forced expression of miR-17-5p. In contrast, the migration and invasion of ovarian cancer cells were decreased when ovarian cancer cells were treated with anti-miR-17-5p inhibitor. Moreover, we found that the expression level of EMT biomarkers was changed in ovarian cancer cells following aberrant expression of miR-17-5p. These results indicate that miR-17-5p plays important role in ovarian cancer progression.

PTEN is a tumor suppressor gene with decreased activity reported in many human cancers [26, 27]. The loss and mutation of PTEN lead to hyperactive PI3K signaling, which is an important intracellular signaling pathway that regulate many cellular processes, including cell survival, cell proliferation, and cell growth. Studies have found that miR-26a acts as a direct regulator of PTEN expression in high-grade glioma [28]. Some miRNAs suppress PTEN expression by directly interacting with its 3′ UTR in prostate epithelial and cancer cells [29]. In the present study, we reported that the expression of PTEN was decreased and the expression of pAKT was increased in ovarian cancer cells. Our results indicate that miR-17-5p affects drug-resistance, apoptosis and invasion by regulating PTEN/Akt signaling pathway in ovarian cancer.However, no miR-17-5p target site in PTEN was identified. Amplified in breast cancer 1 (AIB1) amplification and overexpression have been seen in breast and ovarian cancer cell lines. AIB1 is a steroid receptor coactivator that mediates the transcriptional activities of nuclear receptors and other transcription factors. Hossain et al. has shown that miR-17-5p regulates breast cancer cell proliferation by targeting AIB1 [30]. PTEN can suppress AIB1 through decreasing protein stability [31]. In our study, miR-17-5p affects the PTEN expression in OVCAR-3 cells and SKOV3 cells. Further study should be done to examine the interaction between PTEN and AIB1 in these ovarian cancer cell lines.

## Conclusion

miR-17-5p plays important role in the regulation of tumorigenesis and malignant progression in ovarian cancer. miR-17-5p could be a potential molecular target in ovarian cancer treatment in the future.

## Methods

### Cell lines and cell culture

The human nasopharyngeal carcinoma cell lines, OVCAR-3 and SKOV-3, obtained from American Type Culture Collection were cultured in RPMI1640 medium (Invitrogen, USA) supplemented with 10 % heat-inactivated fetal bovine serum and 100 U ml^−1^ of penicillin and 100 μg ml^−1^ of streptomycin (Sigma, USA). The cells were cultured at 37 °C in a humidified incubator in an atmosphere of 5 % CO_2_-95 % air. All cells were passaged when they reached approximately 80 % confluency.

Transfection miR-17-5p mimics and anti-miR-17-5p inhibitor (single-stranded, modified RNA molecule) were purchased from GenePharma (Shanghai, China). When OVCAR-3 and SKOV-3 cells reached 70 % confluency, they were transfected with miR-17-5p or anti-miR-17-5p inhibitor using Lipofectamine 2000 (Invitrogen, USA) according to the manufacturer’s instructions. The miRNA-Lipofectamine 2000 complex was made in serum-free OPTI-MEM medium. The scrambled oligonucleotide was used as a negative control.

### Real-time RT-PCR quantification of miR-17-5p

To examine the miR-17-5p expression after transfection, total RNA was extracted with mirVanamiRNA Isolation kit (Ambion, USA) according to the manufacturer’s instruction. cDNA was synthesized from the isolated RNA with Taqman MicroRNA Reverse Transcription kit (Thermofisher Scientific, USA). The PCR condition used was: 95 °C for 6 min, followed by 35 cycles of 95 °C for 35 s, 60 °C for 30 s and 72 °C for 30 s, and a dissociation stage. PCR was performed using the TaqMan Fast Universal PCR Master Mix (Thermofisher Scientific, USA) and CFX Connect Real-Time PCR Detection System (Bio-Rad, USA). The endogenous reference gene GAPDH was used for RNA quantification. The PCR primers sequences used were: 5′-GTCTCCTCTGACTTCAACAGCG-3′ and 5′-ACCACCCTGTTGCTGTAGCCAA-3′ (GAPDH).

### The effect of miR-17-5p on cell proliferation after chemotherapy reagent treatment

To evaluate the effect of miR-17-5p on cell proliferation after chemotherapy reagent treatment, the MTT (3-(4,5-Dimethylthiazol-2-yl)-2,5-Diphenyltetrazolium Bromide) assay was performed as described previously [32]. Briefly, OVCAR-3 and SKOV-3 cells, transfected with either scrambled oligonucleotide or miR-17-5p mimic, were seeded in triplicate to 96-well plates at the density of 2 × 10^4^ cells well^−1^. After overnight growth, culture medium containing paclitaxel (6 µmol L^−1^) (Sigma, USA) was added. On every each other day, MTT solution (20 μl, 5 mg ml^−1^) was added to each well, and the plates were incubated in the dark for 4 h at 37 °C, followed by removal of the culture medium and addition of 100 μl of dimethyl sulphoxide (DMSO). The absorbance was measured at 490 nm, with 650 nm as the reference wave length. All experiments were carried out in triplicates.

### Caspase 3/7 activity

OVCAR-3 and SKOV-3 cells, transfected with either scrambled oligonucleotide or miR-17-5p mimic, were seeded in 24-well plates at a density of 1 × 10^5^ cells well^−1^. After overnight incubation in an atmosphere of 5 % CO_2_-95 % air, the supernatant was replaced with culture medium containing different concentrations of paclitaxel (3, 6 and 12 µmol L^−1^). The cells were grown for 48 h, then Caspase-Glo reagent (Promega, USA) was added to each well and incubated at room temperature for 8 h with gentle shaking. The caspase 3/7 activity was measured using 1 min lag time and 0.5 s well^−1^ read time with luminometer (Thermofisher Scientific, USA). The experiments were performed in triplicate.

### TUNEL assay

After overexpression of miR-17-5p, OVCAR-3 and SKOV-3 cells were seeded in 96-well plates at density of 1 × 10^4^ cells well^−1^. After overnight incubation, the supernatant was replaced with culture medium containing paclitaxel (12 µmol L^−1^). The cells were grown for another 48 h, then the TUNEL assay was performed using Click-iT^®^ TUNEL Alexa Fluor^®^ Imaging Kit (Invitrogen, USA) in accordance with the manufacturer’s protocol. In brief, after the cells were fixed with 4 % paraformaldehyde in PBS at room temperature for 20 min and permeabilized with Triton X-100 (0.25 % in PBS) for another 20 min, the cells were washed twice and incubated with terminal deoxynucleotidyltransferase reaction buffer for 10 min at room temperature. The TUNEL reaction mixture containing terminal deoxynucleotidyltransferase was added and the samples were incubated in a humidified chamber at 37 °C for 60 min. Then, samples were washed three times with 3 % BSA in PBS for 2 min each and then incubated with Click-iT reaction mixture (containing Alexa 488 azide) for 30 min at room temperature. After washed with 3 % BSA in PBS, the cell nuclei were counter stained with Hoechst 33342 (Thermofisher Scientific, USA) for 15 min at room temperature. The TUNEL-positive cells were counted in eight different, random fields for each well.

### Matrigel invasion assays

The cell invasion was examined by Matrigel invasion assays according to the manufacturer’s instruction (Promega, USA). Briefly, OVCAR-3 and SKOV-3 cells at density of 3 × 10^4^ per well, transfected with either miR-17-5p mimic or anti-miR-17-5p inhibitor, were placed to the upper BD Biocoat Matrigel Invasion Chamber (BD Bioscience, US) in 0.5 ml DMEM with 0.1 % BSA. The DMEM medium containing 5 % FBS was added to the lower chamber. The cells were incubated for 18 h, and then the non-invaded cells were removed with a cotton swab. The invaded cells were stained by Diff Quik stain (Thermofisher Scientific, USA) and counted under microscopy. The percentage of invasion was expressed as the ratio of invading cells over cell number normalized on day 2 of the growth curve.

### Western blot assay

The transfected OVCAR-3 and SKOV-3 cells were lysed with ice-cold RIPA buffer (Beyotiem, China). Then the samples were mixed with ×6 loading buffer, boiled at 100 °C for 5 min, transferred on ice and loaded to an SDS-PAGE gel. Proteins were separated by SDS-PAGE and transferred to PVDF membranes (Sigma, USA). Then the membranes were incubated in 5 % non fat dry milk in Tris-buffered saline Tween-20 buffer (TBST: 10 mmol L^−1^ Tris-Base, 150 mmol L^−1^ NaCl, 0.05 % Tween-20; pH 7.4) for 1 h at room temperature to block nonspecific antibody binding sites. After washing with TBST buffer, membranes were incubated overnight at 4 °C with primary antibodies (E-cadherin, N-cadherin, Snail, Vimentin, Bcl-2, Bax, AKT and PTEN: Cell Signaling Technology, USA) in TBST with gentle agitation. After washed with TBST at room temperature, the membranes were incubated with the horseradish peroxidase-conjugated secondary antibody for 1 h at room temperature. The immune blot signals were visualized using the EasySeeWeatern Blot Kit (Transgen, China). The protein bands were detected by densitometric scanning (Tanon-1600 gel image system, Shanghai, China).

### Statistical analysis

All of results were shown as mean ± SD. Statistical analyses were performed by Student’s *t* test. Briefly, the experimental results from control groups and experimental groups were entered in SPSS program (version 11.0, IBM. USA), the *p* values were calculated. Differences are considered statistically significant at *p* < 0.05.

## References

[1] Mezzanzanica D (2015). Ovarian cancer: a molecularly insidious disease. Chin J Cancer..

[2] Gasiorowska E, Michalak M, Warchoł W, Lemańska A, Jasiński P, Spaczyński M (2015). Clinical application of HE4 and CA125 in ovarian cancer type I and type II detection and differential diagnosis. Ginekol Pol.

[3] Ahmad B, Nawaz S, Ali S, Bashir S, Mahmood N, Gul B (2015). Level and evaluation of tumor marker CA-125 in ovarian cancer patients in Khyber Pakhtunkhwa, Pakistan. Asian Pac J Cancer Prev.

[4] Liu X, Gao Y, Lu Y, Zhang J, Li L, Yin F (2015). Oncogenes associated with drug resistance in ovarian cancer. J Cancer Res Clin Oncol.

[5] Zou J, Yin F, Wang Q, Zhang W, Li L (2015). Analysis of microarray-identified genes and microRNAs associated with drug resistance in ovarian cancer. Int J Clin Exp Pathol..

[6] Zhang S, Lu Z, Unruh AK, Ivan C, Baggerly KA, Calin GA (2015). Clinically relevant microRNAs in ovarian cancer. Mol Cancer Res.

[7] Chong GO, Jeon HS, Han HS, Son JW, Lee YH, Hong DG (2015). Differential microRNA expression profiles in primary and recurrent epithelial ovarian cancer. Anticancer Res.

[8] Ibrahim FF, Jamal R, Syafruddin SE, Ab Mutalib NS, Saidin S, MdZin RR (2015). MicroRNA-200c and microRNA-31 regulate proliferation, colony formation, migration and invasion in serous ovarian cancer. J Ovarian Res..

[9] Hu X, Macdonald DM, Huettner PC, Feng Z, El Naqa IM, Schwarz JK (2009). A miR-200 microRNA cluster as prognostic marker in advanced ovarian cancer. Gynecol Oncol.

[10] Llauradó M, Majem B, Altadill T, Lanau L, Castellvi J, Sánchez-Iglesias JL (2014). MicroRNAs as prognostic markers in ovarian cancer. Mol Cell Endocrinol.

[11] Hong F, Li Y, Xu Y, Zhu L (2013). Prognostic significance of serum microRNA-221 expression in human epithelial ovarian cancer. J Int Med Res.

[12] Lu J, Getz G, Miska EA, Alvarez-Saavedra E, Lamb J, Peck D (2005). MicroRNA expression profiles classify human cancers. Nature.

[13] Garzon R, Fabbri M, Cimmino A, Calin GA, Croce CM (2006). MicroRNA expression and function in cancer. Trends Mol Med..

[14] Wang D, Qiu C, Zhang H, Wang J, Cui Q, Yin Y (2010). Human microRNA oncogenes and tumor suppressors show significantly different biological patterns: from functions to targets. PLoS One.

[15] Liu X, Chen X, Yu X, Tao Y, Bode AM, Dong Z (2013). Regulation of microRNAs by epigenetics and their interplay involved in cancer. J Exp Clin Cancer Res..

[16] Li L, He L, Zhao JL, Xiao J, Liu M, Li X (2015). MiR-17-5p up-regulates YES1 to modulate the cell cycle progression and apoptosis in ovarian cancer cell lines. J Cell Biochem.

[17] Zeng X, Xiang J, Wu M, Xiong W, Tang H, Deng M (2012). Circulating miR-17, miR-20a, miR-29c, and miR-223 combined as non-invasive biomarkers in nasopharyngeal carcinoma. PLoS One.

[18] Cloonan N, Brown MK, Steptoe AL, Wani S, Chan WL, Forrest AR (2008). The miR-17-5p microRNA is a key regulator of the G1/S phase cell cycle transition. Genome Biol.

[19] Ulker V, Kuru O, Numanoglu C, Akbayir O, Polat I, Uhri M (2014). Lymph node metastasis in patients with epithelial ovarian cancer macroscopically confined to the ovary: review of a single-institution experience. Arch Gynecol Obstet.

[20] Zhang W, Yang YZ, Li L, Wang Q (2013). Study of growth, invasion and metastasis on ovarian epithelial cancer cell line with CCL18 over-expression by mediated in vitro. Zhonghua Fu Chan Ke Za Zhi.

[21] Tsai JH, Yang J (2013). Epithelial-mesenchymal plasticity in carcinoma metastasis. Genes Dev.

[22] Kalluri R, Weinberg RA (2009). The basics of epithelial-mesenchymal transition. J Clin Invest..

[23] Zhou X, Hu Y, Dai L, Wang Y, Zhou J, Wang W (2014). MicroRNA-7 inhibits tumor metastasis and reverses epithelial-mesenchymal transition through AKT/ERK1/2 inactivation by targeting EGFR in epithelial ovarian cancer. PLoS One.

[24] Jin M, Yang Z, Ye W, Xu H, Hua X (2014). MicroRNA-150 predicts a favorable prognosis in patients with epithelial ovarian cancer, and inhibits cell invasion and metastasis by suppressing transcriptional repressor ZEB1. PLoS One.

[25] Chen D, Zhang Y, Wang J, Chen J, Yang C, Cai K (2013). MicroRNA-200c overexpression inhibits tumorigenicity and metastasis of CD117^+^CD44^+^ ovarian cancer stem cells by regulating epithelial-mesenchymal transition. J Ovarian Res..

[26] Andjelkovic T, Bankovic J, Milosevic Z, Stojsic J, Milinkovic V, Pesic M, et al. Concurrent alteration of p16 and PTEN tumor suppressor genes could be considered as potential molecular marker for specific subgroups of NSCLC patients. Cancer Biomark. 2011–2012;10:277–86. doi:10.3233/CBM-2012-0257. 10.3233/CBM-2012-0257 PMC13016250 22820083

[27] Stewart DJ, Nunez MI, Jelinek J, Hong D, Gupta S, Aldaz M (2014). Impact of decitabine on immunohistochemistry expression of the putative tumor suppressor genes FHIT, WWOX, FUS1 and PTEN in clinical tumor samples. Clin Epigenet.

[28] Huse JT, Brennan C, Hambardzumyan D, Wee B, Pena J, Rouhanifard SH (2009). The PTEN-regulating microRNA miR-26a is amplified in high-grade glioma and facilitates gliomagenesis in vivo. Genes Dev.

[29] Tian L, Fang YX, Xue JL, Chen JZ (2013). Four microRNAs promote prostate cell proliferation with regulation of PTEN and its downstream signals in vitro. PLoS One.

[30] Hossain A, Kuo MT, Saunders GF (2006). Mir-17-5p regulates breast cancer cell proliferation by inhibiting translation of AIB1 mRNA. Mol Cell Biol.

[31] Yang C, Li S, Wang M, Chang AK, Liu Y, Zhao F (2013). PTEN suppresses the oncogenic function of AIB1 through decreasing its protein stability via mechanism involving Fbw7 alpha. Mol Cancer..

[32] Maioli E, Torricelli C, Fortino V, Carlucci F, Tommassini V, Pacini A (2009). Critical appraisal of the MTT assay in the presence of rottlerin and uncouplers. Biol Proced Online..

